# Simultaneous VO_2_ and cardiac output measurement to estimate oxygen extraction (a-v)O_2_

**DOI:** 10.1186/1532-429X-18-S1-W39

**Published:** 2016-01-27

**Authors:** Richard A LaFountain, Juliet Varghese, Juliana Serafim da Silveira, Debbie Scandling, Orlando P Simonetti

**Affiliations:** 1 grid.261331.40000000122857943Biomedical Engineering, The Ohio State University, Columbus, OH USA; 2 grid.261331.40000000122857943Human Sciences, The Ohio State University, Columbus, OH USA; 3 grid.261331.40000000122857943Davis Heart & Lung Institute, The Ohio State University, Columbus, OH USA; 4 grid.261331.40000000122857943Internal Medicine, The Ohio State University, Columbus, OH USA

## Background

Chronic heart failure (CHF) is the leading hospital discharge diagnosis in patients over age 65 [1]. Emerging techniques in cardiovascular magnetic resonance (CMR) have resulted in unique opportunity for improvement of non-invasive assessment of the physiologic and anatomic effects of CHF. We have previously demonstrated the accuracy and feasibility of ${\dot{\text{V}}}_{\text{O}}2\max$ measurements in the MRI environment using a modified metabolic cart [2]. Additionally, ${\dot{\text{V}}}_{\text{O}}2$ measures acquired within the MRI have been reported [3]. Existing methods describe non-invasive MRI measurement of whole body oxygen consumption via T2 imaging [4]. The Fick principle states ${\dot{\text{V}}}_{\text{O}}2$ = CO × (A-V)O_2;_ where ${\dot{\text{V}}}_{\text{O}}2$ is oxygen consumption, CO is cardiac output, and (A-V)O_2_ is arterio-venous oxygen difference. Current modifications required for metabolic cart measures of oxygen consumption in the MRI environment present clinical challenges in widespread application. Using the Fick principle we sought to quantify and compare non-invasive MRI derived ${\dot{\text{V}}}_{\text{O}}2$ (MRI-${\dot{\text{V}}}_{\text{O}}2$) quantification with metabolic cart measures of ${\dot{\text{V}}}_{\text{O}}2$.

## Methods

Eight subjects underwent CMR exam with in-magnet ${\dot{\text{V}}}_{\text{O}}2$ measures via ParvoMedics TrueOne metabolic cart. A 21 foot hose with non-rebreathing mask was passed through a waveguide for measurement of ${\dot{\text{V}}}_{\text{O}}2$ (L/min); single breath analysis with 22 second delay was used to evaluate ${\dot{\text{V}}}_{2}\text{O}$ during CMR measures. CO (L/min) was quantified using real time aortic flow imaging allowing beat-by-beat stroke volume quantification. Heart rate was averaged over the duration of flow imaging sequence to reduce noise. Oxygen saturation was used to calculate (A-V)O_2_ (ml O_2_/100ml blood) from quantitative T2 mapping of the left and right ventricle blood pools. The Fick principle was used to quantify ${\dot{\text{V}}}_{\text{O}}2$ derived from MRI measures of CO and (A-V)O_2_ for comparison with measured VO_2_. Lin's Concordance Correlation Coefficient (ρ_c_) and the 95 percent lower confidence level (95% LCL) were used to evaluate agreement between two techniques MRI-${\dot{\text{V}}}_{\text{O}}2$ and metabolic cart ${\dot{\text{V}}}_{\text{O}}2$ measures.

## Results

**Figure 1:**
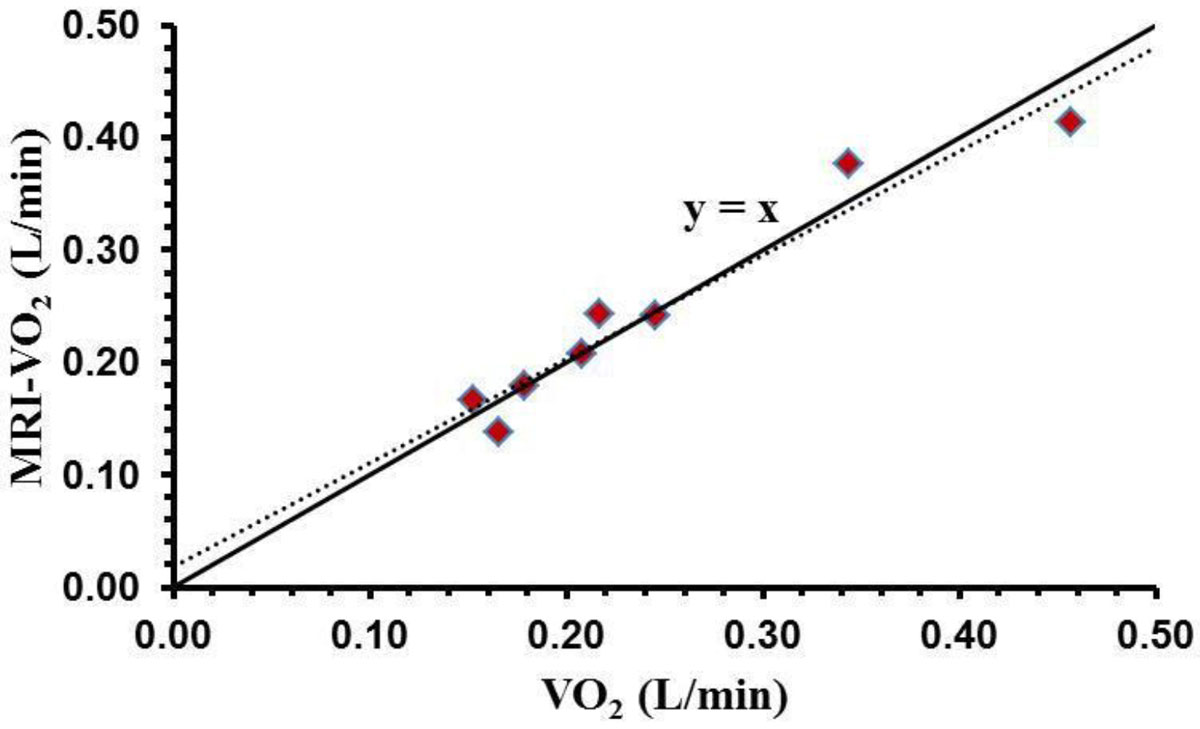
Figure 1

Mean ${\dot{\text{V}}}_{\text{O}}2$ measures from the metabolic cart and MRI-VO2 shown in Figure 1. MRI-${\dot{\text{V}}}_{\text{O}}2$ vs ${\dot{\text{V}}}_{\text{O}}2$ Concordance Correlation Coefficient Plot were within reasonable limits for supine resting measures (0.15-0.46 L/min). Figure 1 depicts measured ${\dot{\text{V}}}_{\text{O}}2$ versus MRI-${\dot{\text{V}}}_{\text{O}}2$ for each of eight subjects. ρ_c_ was 0.9669, while the 95% LCL was 0.8815.

## Conclusions

Non-invasive MRI derived ${\dot{\text{V}}}_{\text{O}}2$ with flow imaging quantification of CO and quantitative T2 mapping was shown to be feasible and interchangeable (95% LCL >0.75) with measured ${\dot{\text{V}}}_{\text{O}}2$ [5]. Further investigation is needed to validate these results against independent, catheter-based measurement of venous O_2_ saturation. The combination of VO_2_ and CMR will potentially enhance non-invasive estimation of oxygen extraction, and when combined with exercise stress may be helpful in determining whether cardiac function or peripheral oxygen extraction, are the primary cause of heart failure related symptoms in individual patients.

